# Evaluating vaginal moisture in mice with calibrated filter paper

**DOI:** 10.21203/rs.3.rs-5953014/v1

**Published:** 2025-02-06

**Authors:** Mohan Liu, Joseph G Charek, Rose Kurian, Rodolfo D Vicetti Miguel, Thomas L Cherpes

**Affiliations:** The Ohio State University College of Medicine; The Ohio State University College of Medicine; The Ohio State University College of Medicine; The Ohio State University College of Medicine; The Ohio State University College of Medicine

**Keywords:** calibrated filter paper strips, DMPA, ovariectomized mice, vaginal moisture

## Abstract

**Objective:**

Loss of ovarian estrogen (E) production in postmenopausal women causes vaginal dryness and irritation. The contraceptive depot-medroxyprogesterone acetate (DMPA) likewise promotes vaginal dryness by suppressing ovulation and reducing E production. While vaginal dryness in women is identified by self-report, mouse models of estrogen loss require methods are developed to objectively measure vaginal moisture.

**Methods:**

Strips of calibrated filter paper, similar to those used to quantify lachrymal secretions, were intravaginally placed in untreated ovary-intact mice, DMPA-treated ovary-intact mice, and ovariectomized (OVX) mice to measure vaginal fluid levels.

**Results:**

Median readings from calibrated filter paper strips from DMPA-treated and OVX mice were significantly lower than the median reading from estrus-stage mice. Levels of circulating estradiol were also significantly lower in DMPA-treated and OVX mice vs. estrus-stage mice.

**Conclusions:**

Calibrated filter paper provides objective measure of vaginal moisture in mice. Current findings also indicate that hypoestrogenemic mice (DMPA-treated or OVX) accurately model the vaginal moisture losses identified in women with lower levels of circulating E.

## INTRODUCTION

Approximately 1.2 billion women worldwide will be menopausal or postmenopausal (PM) by 2030 (1). Most of these women will be affected by the genitourinary syndrome of menopause (GSM) (2), a constellation of signs and symptoms that includes vaginal dryness, dyspareunia, dysuria, and recurrent urinary tract infection (3–5). GSM signs and symptoms occur as the loss of circulating estrogen (E) after menopause causes changes in vaginal tissue that include epithelial thinning, increased connective tissue density, and loss of collagen and elastin (6). These changes reduce vaginal tissue elasticity and enhance its susceptibility to mechanical stress (7). GSM symptoms also impact quality of life by diminishing partner intimacy (8).

The goal for treating GSM is symptomatic relief and options include hydrating agents, hormone therapy, thermo-ablative fractional CO_2_ laser, and radiofrequency energy (9–12). While vaginal dryness commonly occurs in PM women, clinical research typically does not quantify treatment-induced changes in vaginal moisture. More frequently, clinical research used composite scoring systems to define the effects of GSM treatment. These included the Vaginal Health Index (VHI), which subjectively evaluates vaginal fluid volume observed with speculum exam (13) and the Vaginal Maturation Index (VMI), which calculates the proportion of parabasal, intermediate, and superficial cells in vaginal smears (13). Smears that contain more parabasal cells and fewer superficial cells are characteristic of the vulvovaginal atrophy (VVA) that develops after onset of menopause (14). In addition to the VHI and VMI, clinical trials used participant questionnaires, including the Vulvovaginal Symptoms Questionnaire, the Day-to-Day Impact of Vaginal Aging Questionnaire, and Vaginal and Vulvar Assessment Scale to evaluate treatment efficacy (15). Similar to the use of composite scoring systems, however, responses to these questionnaires did not objectively measure treatment-induced changes in vaginal moisture.

On the other hand, a 2022 publication described the use of intravaginally placed calibrated filter paper strips, like those used to quantify lachrymal secretions, to measure vaginal moisture (16). These strips absorb fluid by capillary action (with rates of fluid travel along the strip proportional to rates of fluid production). In this 2022 report, intravaginally placed strips identified significantly lower moisture levels in PM women with GSM vs. women without vaginal dryness (16). These results newly identified an objective measure of vaginal moisture and suggested this assay will benefit assessment of vaginal dryness in both PM women and women using the contraceptive depot-medroxyprogesterone acetate (DMPA). Similar to the loss of ovarian E production after menopause, DMPA causes hypoestrogenemia that promotes vaginal dryness (17). Herein, we explored using calibrated filter paper strips to quantify vaginal moisture in mice with diminished E production and normal controls. Specifically using these strips to compare vaginal moisture levels in ovary-intact mice, DMPA-treated ovary-intact mice, and ovariectomized (OVX) mice.

## MATERIALS AND METHODS

### Mice

All study procedures complied with American Association for Accreditation of Laboratory Animal Care regulations and were approved by The Ohio State University (OSU) Institutional Animal Use and Care Committee. For these studies, 8–10-week-old ovary-intact and OVX mice were purchased from Jackson Laboratories (Bar Harbor ME, USA). Where indicated, ovary-intact mice (hereafter termed intact mice) were subcutaneously (SQ) injected with 0.3 mg of DMPA (Mylan Institutional, Rockford IL USA) suspended in 100 μl of phosphate buffered saline (PBS) (Mediatech Inc., Manassas VA USA).

### Vaginal moisture measurements

**Figure 1:**
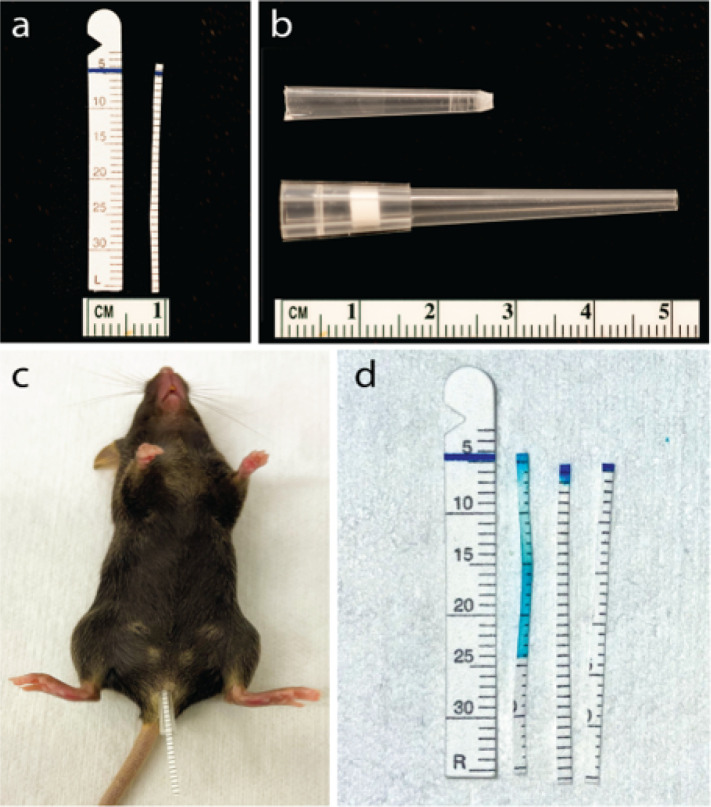
Quantifying vaginal moisture in mice. a) Commercially available calibrated filter paper strips were trimmed for intravaginal (ivag) insertion. b) To ensure atraumatic ivag insertion of these strips, 200 μL wide-bore pipette tips were shortened to a 2.5 cm length and openings of the tips filed for smoothness. c) Intravaginally inserted paper strips were retained for 5 minutes in sedated mice. d) Representative results depict vaginal fluid levels as visualized by migration of the blue line from its original position by capillary action.

Calibrated filter paper strips (Merck Animal Health, Summit NJ USA) were cropped to a width of 1 mm and horizontally cut near the blue indicator line used to visualize fluid absorption (Fig. 1a). To ease intravaginal (ivag) insertion of modified calibrated filter paper strips, 200 μL wide-bore pipette tips (Thermo Fisher Molecular BioProducts, Pittsburgh PA USA) were trimmed 2.5 cm lengths and tip openings filed for smoothness (Fig. 1b). To ensure atraumatic ivag insertion, pipettes were flushed with distilled water (Mediatech Inc.) and smoothness of the tip opening confirmed by optical microscope exam (Leica Microsystems, Deerfield IL USA). Mice were sedated with 1.8 mg of ketamine hydrochloride (JHP Pharmaceuticals, Rochester MI USA) and 0.18 mg of xylazine (Lloyd Laboratories, Shenandoah IA USA). First, pipette tips were inserted in sedated mice (to simplify strip insertion and avoid urine contact) and the paper strips (with calibration marks visible) advanced through the tip to contact cervicovaginal mucosa (Fig. 1c). Mice were placed in dorsal recumbency and paper strips retained in this position for 5 minutes. Strips were removed and blue line migration from its original position recorded (Fig. 1d). Vaginal moisture levels were quantified in OVX mice, mice in various estrous cycle stages, and 5 days after intact mice were administered 0.3 mg of DMPA. Investigators measuring vaginal moisture levels were unaware of experimental group assignments. At the time vaginal fluid levels were evaluated, peripheral blood samples were collected to quantify serum estradiol levels by ELISA (ALPCO, Salem NH USA) (limit of detection 3 pg/mL) (18).

### Cervicovaginal lavage (CVL) collection

Prior to vaginal fluid measurements, CVL were collected from DMPA-treated intact mice and OVX mice for 3 consecutive days to confirm absence of estrous cycling. CVL were also collected from untreated intact mice after calibrated filter paper strip were removed to identify estrous cycle stage. Cells collected by CVL were crystal violet-stained and examined with an inverted light microscope (Laxco Inc., Mill Creek WA USA). While most cells from estrus-stage mice are cornified squamous epithelial cells in densely packed clusters, cells from metestrus-stage or diestrus-stage mice are mainly leukocytes with lower frequency of cornified squamous epithelial cells (19). Distinction between metestrus and diestrus estrous cycle stages was made upon identification of nucleated epithelial cells in the latter (19).

### Statistical considerations

All analyses were performed using Prism 10 software (GraphPad, La Jolla CA USA). Moisture values were acquired from individual mice. For multiple comparisons, we used the Kruskal-Wallis test with Dunn’s test for multiple comparisons. *P* values less than 0.05 were deemed statistically significant.

## RESULTS

**Figure 2:**
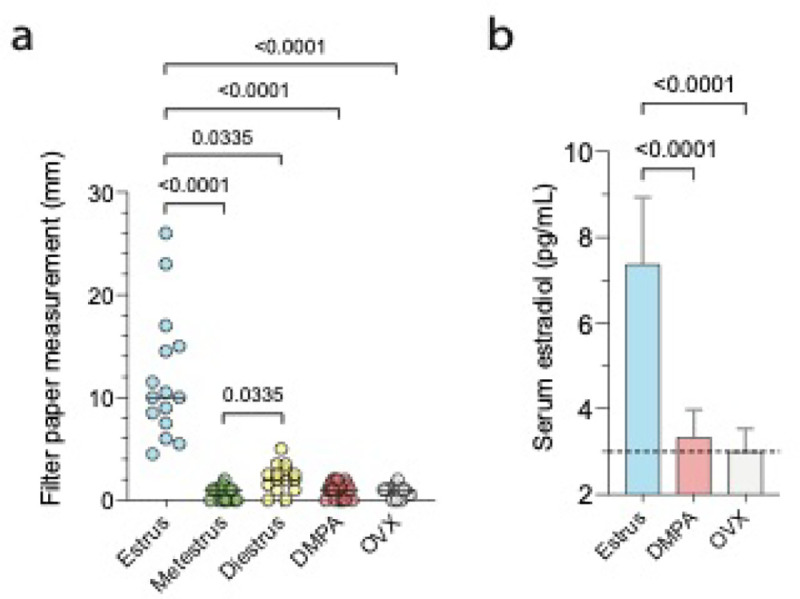
Vaginal fluid levels were significantly higher in estrus-stage mice vs. diestrus-stage, metestrus-stage, DMPA-treated, or OVX mice. A) A median value of 10.0 mm was calculated from calibrated filter paper strips readings collected from estrus-stage mice (n = 15) while median readings of 1.0 mm,2.0 mm, 1.0 mm, and 1.0 mm , respectively, were recorded from strips placed in metestrus-stage (n = 15), diestrus-stage (n = 15), DMPA-treated (n = 20) and OVX mice (n = 15). B) Serum estradiol levels were comparable between DMPA-treated (n = 15) and OVX mice (n = 15) and significantly lower than values measured in untreated mice in the estrus stage of the estrous cycle (n = 15). DMPA, depot-medroxyprogesterone acetate; OVX, ovariectomized.

After developing methodology that allowed us to use calibrated filter paper strips to quantify vaginal fluid in mice (Fig. 1), we compared levels of vaginal fluid from intact mice in the estrus, metestrus, or diestrus stages of the estrous cycle. For this study, we posited that vaginal fluid levels are higher in mice in a sexually receptive phase (i.e., estrus) vs. a sexually non-receptive phase (i.e., metestrus or diestrus) (20). To test this hypothesis, moisture readings were recorded from intravaginally placed calibrated filter paper strips and CVL collected to exam crystal-violet-stained vaginal cells for estrus cycle stage identification (19). Both vaginal fluid measurements and CVL were obtained from mice until equal-sized groups of mice in estrus, metestrus, and diestrus were identified (n = 15 per group). As posited, vaginal fluid levels were significantly higher in estrus-stage mice vs. mice in the metestrus or diestrus stages of the estrous cycle (Fig. 2A).

We likewise hypothesized that compared to mice in the estrus stage of the estrous cycle, there are lower levels of vaginal fluid in mice with lower levels of circulating E. To test this hypothesis, we collected moisture readings from OVX mice that model the loss of E in menopausal and PM women (21) and mice injected with DMPA, a progestin-only contraceptive that reduces ovarian E production by suppressing HPO axis function (22). Prior to vaginal fluid quantification, CVL were collected 3 consecutive days from DMPA-treated and OVX mice to confirm absence of estrous cycling. At the time vaginal fluid readings were acquired from the estrus-stage, DMPA-treated, and OVX mice, peripheral blood was collected to quantify serum levels of estradiol. As hypothesized, vaginal fluid levels were significantly higher in estrus-stage mice (median value = 10.0 mm) vs. DMPA-treated intact mice (median value = 1.0 mm) or OVX mice (median value = 1.0 mm) (Fig. 2A). Moreover, serology results identified that the levels of circulating E were significantly lower in DMPA-treated and OVX mice vs. mice in the estrus stage of the estrous cycle (P < 0.001 for both comparisons) (Fig. 2B).

## DISCUSSSION

Consistent with the 2022 publication that used calibrated filter paper strips to detect significantly lower levels of vaginal moisture in PM women with GSM vs. women without vaginal dryness (16), our investigation developed methodology that identified significantly lower levels of vaginal moisture in DMPA-treated and OVX mice vs. estrus-stage mice. As expected, this investigation also detected significantly lower levels of serum E in DMPA-treated and OVX mice. Together, our results indicate that hypoestrogenemia induced by ovariectomization or progestin treatment models the loss in vaginal moisture in PM women and reproductive age women using DMPA for contraception (23, 24). Our results also imply that use of calibrated filter paper strips provides a readily obtained and objective measure of vaginal moisture in mice that will allow research to newly define the efficacy of therapies now used to relieve vaginal dryness. As example, while dehydroepiandrosterone is a U.S. Food and Drug Administration-approved treatment for GSM-related dyspareunia, neither preclinical nor clinical research objectively defined the ability of this drug to restore vaginal moisture (25). Based on the public health significance of menopause, the negative impact of GSM on quality of life for PM women, and absence of preclinical research that defined the ability of GSM therapies to relieve vaginal dryness, calibrated filter paper strips seem likely to provide a practical new tool to objectively quantify vaginal moisture in mice.

Because levels of serum E and vaginal moisture are similarly reduced in DMPA-treated and OVX mice (Fig. 2), the current study indicates that the production of vaginal moisture in mice is an E-dependent process. This possibility is congruent with our previously reported results that identified that vaginal epithelial integrity and barrier function are compromised in OVX and DMPA-treated mice by lack of circulating E (26, 27). Specifically, we showed that compared to mice in the estrus stage of the estrous cycle, vaginal epithelium from OVX and DMPA-treated mice displays significantly lower levels of cell-cell adhesion molecules desmoglein-1 (DSG1) and desmocollin-1 (DSC1) needed for epithelial integrity and significantly less ability to restrict low molecular weight (LMW) molecule entry into subepithelial tissue (26,27). Identification that calibrated filter paper strips offer reliable and objective measure of vaginal fluid in mice, thus complements methodology that defined vaginal epithelial integrity (by quantifying desmosomal proteins DSG1 and DSC1 levels) and barrier protection (by quantifying depth of LMW molecule penetration into subepithelial tissue) and expands our ability to assess vaginal health in mice.

In conclusion, this small-scale study in mice identifies methodology with the potential to newly inform reproductive health research. Though menopause is condition that impacts 1 billion women worldwide, we know remarkably little about the effects of menopause onset on vaginal epithelial biology in general or the specific ability of GSM treatments to restore normal levels of vaginal epithelial moisture. On the other hand, we do know that nearly half of PM women are adversely impacted by GSM and that vaginal dryness is among the most frequent complaints. Composite scores from the VMI and VHI and individual responses to the Vaginal and Vulvar Assessment Scale and other questionnaires indirectly assess menopause-related vaginal dryness, but do not provide objective measure of vaginal moisture. This situation does not diminish the importance of clinical investigation, but does enhance the utility of experimental models that objectively delineate vaginal epithelial health. This includes the capacity to use mice to define the effects of treatments used clinically to restore vaginal moisture. By developing methods that quantify the loss of vaginal moisture in DMPA-treated and OVX mice, results from the current study support the use of mice to newly define E-mediated regulation of vaginal epithelial health and to identify current and candidate treatments that best restore this health.
